# Impact of increasing dietary standardized ileal digestible arginine to lysine ratio from 0.85 to 1.15 and water-based arginine supplementation on growth performance and gut integrity of weaned pigs

**DOI:** 10.1093/tas/txae102

**Published:** 2024-07-10

**Authors:** Chloe Hagen, Dalton Humphrey, Caitlyn Wileman, Keith Haydon, Laura Greiner

**Affiliations:** Department of Animal Science, Iowa State University, Ames, IA 50011, USA; Department of Animal Science, Iowa State University, Ames, IA 50011, USA; Department of Animal Science, Iowa State University, Ames, IA 50011, USA; Department of Animal Science, Iowa State University, Ames, IA 50011, USA; CJ America – Bio, Fort Dodge, IA 50501, USA; Department of Animal Science, Iowa State University, Ames, IA 50011, USA

**Keywords:** arginine, lysine, nursery pigs, weaned pigs

## Abstract

The objective of this experiment was to assess the influence of arginine (Arg) supplementation in water and/or feed on the growth performance and gastrointestinal health of newly weaned pigs. Two hundred and forty pigs (5.06 kg; PIC, Hendersonville, TN) were randomly allocated into 80 mixed-sex pens (3 pigs/pen) and subjected to a 2 × 4 factorial design. Two levels of Arg were supplemented in water (0% or 8% stock, dosed through a 1:128 proportioner) for the first phase (days 0 to 7), and four dietary arginine levels (0.85, 0.95, 1.05, and 1.15) standardized ileal digestible (SID) Arg to Lysine (Lys) ratios for the first two phases (days 0 to 7 and 7 to 21). All treatments were provided a common diet (0.96 SID Arg:Lys) for the last phase days 21 to 42. One pig per pen underwent a dual sugar absorption test of lactulose at 500 mg/kg and mannitol at 50 mg/kg of body weight (BW) via gastric tube on days 7 and 21 postweaning, with blood plasma collected 4 h later. The pig tested on day 7 was subsequently euthanized for intestinal tissue collection. Pen growth performance and feed disappearance were evaluated for 3 phases: days 0 to 7, 7 to 21, and 21 to 42 postweaning. The statistical analysis used linear models to examine the effects of SID Arg:Lys in the feed, Arg level in water, and their interactions, with pen as the experimental unit. Orthogonal contrasts were used to test the linear and quadratic effects of increasing SID Arg:Lys in the diet. Growth performance during the first period exhibited variability, reflected by negative gain-to-feed (G:F) ratios, caused by the enteric health challenge. Consequently, data were analyzed separately for each phase. Increasing dietary SID Arg:Lys caused a linear improvement (*P* = 0.04) in final BW (18.47 and 21.90 kg, for 0.85 and 1.15 SID Arg:Lys, respectively). A trend (*P* = 0.09) suggested a linear impact of dietary SID Arg:Lys on average daily gain during days 21 to 42. Arg supplementation, whether administered through water or diet, did not affect lactulose and mannitol absorption on both days 7 and 21, nor did it alter histological measurements in the collected ileum tissues on day 7 postweaning. In conclusion, increasing dietary SID Arg:Lys increased final BW but had no clear impacts on intestinal health within the parameters measured, potentially impacted by the rotavirus diagnosis in the first week post-wean.

## Teaser

This study investigated how arginine supplementation impacts the growth and gut health of newly weaned pigs in a 42-d study involving 240 animals under a natural enteric disease challenge. The results indicated promising improvements in growth by increasing dietary arginine; however, supplemental arginine did not affect gastrointestinal health under the conditions of this study.

## Introduction

The impact of weaning on piglets is well documented in literature. Weaning represents the most stressful event for a pig documented by changes in diet type (liquid to solid), change in social and physical environment, and pathogen exposure. Many studies have documented the negative impact of weaning on intestinal integrity (Spreeuwenberg et al., 2001; Moeser et al., 2007, 2017; Xun et al., 2018). The disruption to intestinal integrity in the nursery period can reduce nursery growth performance, survivability, and subsequent finishing performance.

Numerous dietary strategies have been explored to mitigate the adverse effects of intestinal damage during the nursery period. One approach involves the incorporation of supplemental dietary l-Arginine (Arg) in nursery diets, either through direct inclusion or water supplementation. l-Arginine is essential in pathways critical for survival such as nitric oxide (NO) synthesis, energy metabolism, polyamine synthesis, cellular protein production, muscle growth, and the synthesis of other functional amino acids. Thus, supplementation of Arg to piglets has shown to increase muscle growth and immunity (Che et al., 2019).

Despite its importance, the levels of circulating Arg decrease in pigs under stress. In the presence of inflammatory stimuli such as cytokines or lipopolysaccharides, enzymatic activity on Arg within the cell increases, resulting in a reduction in plasma Arg levels during infection or inflammation (Li et al., 2007). Notably, Arg bioavailability is diminished during oxidative stress and supplementation demonstrates beneficial effects on Arg metabolism, along with the suppression of inflammatory cytokines (Zheng et al., 2017). This indicates the need for Arg may be higher during times of stress such as the weaning period.

Promising findings were reported by Greiner et al. (2023), indicating that supplementing Arg in nursery diets (SID Arg) during the initial 3 wk led to improvements in final BW. Moreover, water-based Arg supplementation was observed to reduce intestinal permeability. However, considering the competition between lysine (Lys) and Arg for entry into cells along with the negative impact of Lys on arginase activity, it is deemed more appropriate to assess Arg supplementation levels in relation to Lys, as suggested by Wu and Morris (1998).

Building on promising results, a subsequent study examined four dietary Arg levels as a ratio to lysine (SID Arg:Lys) instead of the total diet (SID Arg), along with two water supplementation levels (0% vs. 8%). The aim was to refine the optimal SID Arg:Lys level in early nursery diets, assess water supplementation, and determine if providing extra Arg during this stressful period is beneficial. The hypothesis posited that Arg supplementation through water or diet would enhance growth performance and measurements of intestinal structural integrity and permeability.

## Materials and Methods

### Animal Care Statement

All procedures in this experiment adhered to guidelines for the ethical and humane use of animals for research according to the Guide for the Care and Use of Agricultural Animals in Research and Teaching (FASS, 2010) and were approved by the Institutional Animal Care and Use Committee at Iowa State University (IACUC #22-195).

### Animals and Experimental Design

Two hundred and forty barrows and gilts with an average body weight (BW) of 5.06 kg (PIC 337 × 1,050, PIC Genus, Hendersonville, TN) arrived immediately after weaning at approximately 21 d of age. Pigs were randomly allocated (mixed gender, 3 pigs/pen) into a total of 80 test pens at the Iowa State Swine Nutrition Research Facility (Ames, IA) upon arrival. Room configuration allowed for two different Arg-water treatments with 40 pens per water line. Within each waterline, pens were randomly assigned to a complete randomized design to 1 of the 4 Arg-dietary treatments across the 40 pens. Once allotted into pens, pigs were placed onto the treatment regimens (2 × 4 factorial). Each pen had one single-hole feeder and two nipple drinkers. The pigs were porcine reproductive and respiratory syndrome and porcine epidemic diarrhea virus negative and were vaccinated against *Mycoplasma hyopneumoniae* and porcine circovirus prior to weaning. Upon exhibiting symptoms of disease at day 3 post-placement, uniformly across pens, pigs tested positive for Rotaviral enteritis, Coccidiosis, and Escherichia coli at day 6 post-placement, following analysis at the Iowa State University Veterinary Diagnostic Laboratory (Ames, IA). Subsequently, pigs were treated in accordance with the standard farm protocol using ceftiofur (dosage: 1 mL/20 kg BW, Exceed, Zoetis, Parsippany, NJ), administered by blinded staff.

### Treatments

The study consisted of four dietary treatments (Table 1), which included 0.85, 0.95, 1.05, and 1.15 standardized ileal digestible (SID) Arg to lysine (SID Arg:Lys) ratios. Crystalline arginine (CJ America—Bio, Fort Dodge, IA) was added to the diets at the expense of corn starch, all other ingredients remained the same. For the two levels of water treatment, feed-grade Arg was added to clean plastic buckets filled with fresh water at 0% or 8% stock solution. The 1:128 proportioner (D25 Fixed Dosatron Injector, Dosatron International, Clearwater, FL) was used to distribute the solution into one of the two water lines, resulting in a final concentration of 0% or 0.0625% Arg. This yielded an estimated intake of 0 or 0.44 g of Arg/d/pig, respectively, utilizing the anticipated average daily water intake of 0.7 L/d. All diets were formulated to be isocaloric, with consistent levels of all other ingredients except dietary Arg and corn starch. All nutrient levels exceeded or met NRC (2012) requirements. Upon arrival, pigs were placed on both water and dietary treatments, with water treatments discontinued at the end of phase 1 (day 7). Pigs remained on the dietary Arg treatments throughout phase 2 (day 21), after which they were placed on a common diet (SID Arg:Lys 0.96) for the remaining 21 d of the nursery period.

**Table 1.** Diet composition for the trial to assess arginine levels in nursery pig diets over 42 d

	*Phase 1* *(days 0 to 7)*	*Phase 2* *(days 7 to 21)*	*Phase 3* *(days 21 to 42)*
SID Arg:Lys^*^	0.85	0.85	Common
Corn	32.10	46.67	59.18
SBM (47.5 CP)	20.00	28.00	34.00
Oat groats	15.00	5.00	0.00
DairyLac 80^†^	12.91	5.41	0.00
Fishmeal	5.00	2.50	0.00
Animal plasma	4.61	2.44	0.00
Soy oil	3.00	3.00	3.00
Dried whey	2.50	2.50	0.00
Calcium carbonate	0.81	0.92	0.99
Monocal phosphate	1.24	0.89	0.84
l-lysine HCl	0.40	0.42	0.45
Corn starch	0.44	0.43	0.00
Zinc oxide	0.38	0.38	0.00
Salt	0.30	0.30	0.50
l-methionine	0.26	0.26	0.25
Arginine^‡^	0.22	0.04	0.00
ThrPro^¶^	0.22	0.22	0.24
Vitamin premix^‖^	0.25	0.25	0.25
Trace mineral premix^‖^	0.15	0.15	0.15
l-Valine	0.09	0.11	0.12
l-tryptophan	0.06	0.06	0.04
Copper sulfate	0.07	0.07	0.00
Phytase^$^	0.00	0.00	0.01
Calculated composition			
ME^**^, Mcal/kg	3.31	3.39	3.47
Crude protein, %	22.4	22.1	21.1
Calcium, %	0.93	0.79	0.78
AvP^††^, %	0.6	0.4	0.37
Lactose	12	6	0
SID Arg, %	1.24	1.21	1.27
SID Lys, %	1.46	1.42	1.33
SID Arg:Lys^*^	0.85	0.85	0.96

^*^Increasing SID Arg:Lys levels (0.95, 1.05, and 1.15) achieved by adding arginine at the expense of corn starch in each phase. Phase 3 is a common diet fed to all pigs.

^†^DairyLac 80 is a granular, high-lactose ingredient manufactured through the process of dry rolling liquid whey permeate that contains 3% crude protein and 80% lactose.

^‡^Arg was provided as free base.

^‖^Vitamin and trace mineral; provided 7,656 IU vitamin A, 875 IU vitamin D, 63 IU vitamin E, 3.8 mg vitamin K, 70 mg niacin, 33.8 mg pantothenic acid, 13.8 mg riboflavin, 0.06 mg vitamin B12, 165 mg Zn (zinc sulfate), 165 mg Fe (iron sulfate), 39 mg Mn (manganese sulfate), 16.5 mg Cu (copper sulfate), 0.3 mg I (calcium iodate), and 0.3 mg Se (sodium selenite) per kilogram of diet.

^$^Ronozyme Hi Phos, expected release of 0.12 available phosphorus.

^¶^Threonine source, (CJ America Bio, Fort Dodge, IA).

^**^Metabolizable energy.

^††^Available phosphorus.

SID, standardized ileal digestible; Lys, lysine; Arg, arginine. Ingredients are listed as a percent inclusion in the diet and reported on an as-fed basis.

### Sample Collection and Analysis

Pigs and feeders were weighed at the beginning of the trial, day 7 (water and diet change), day 21 (diet change), and day 42 (end of trial) to calculate average daily gain (ADG), average daily feed intake, and gain to feed ratio (G:F).

On day 7, prior to diet and water treatment change, one pig per pen (closest to average BW of pen) was orally administered a solution of lactulose (500 mg/kg BW) and mannitol (50 mg/kg BW) via a gastric tube at a dose of 1 mL/kg of BW. Four hours post-gavage, blood was collected via a sterile jugular venipuncture using a 10-mL plasma vacutainer tube (EDTA, Becton Dickinson, Franklin Lakes, NJ), inverted, and immediately stored on ice. The blood was separated via centrifugation (2,000 × *g* for 10 min at 4 °C), and the plasma was collected and stored at −80 °C for later analysis of lactulose and mannitol concentration. Following bleeding, these same pigs were euthanized by captive bolt stunning, followed by exsanguination.

Post-euthanasia, the abdomen was opened, and a 2-cm segment of the terminal ileum was dissected, washed with phosphate-buffered saline (pH 7.4), and stored in 10% neutral buffered formalin solution at room temperature for 48 h. After 48 h, tissue samples were moved into 70% ethanol before being submitted to the Iowa State University Diagnostic Laboratory (Ames, IA) for villus and crypt depth evaluation. In short, the formalin-fixed ilea were processed and embedded in paraffin wax. A 5-µ transverse section was cut, stained with hematoxylin and eosin, and mounted on glass slides. Images of ileal sections were taken. Ten well-oriented villi and crypt pairs were selected. The height of each villus was measured from the top of the villus to the crypt transition. The crypt depth was defined as the invagination between two villi. The 10 individual measurements were taken from each pig, and the average of the 10 observations was used in the statistical analysis. A villous height to crypt depth ratio (villous:crypt) was calculated from the measurements of crypt depth and villous height for each sample.

The remaining pigs in the pen were then taken off the Arg-water treatment and continued phase 2 dietary treatments, being the same dietary SID Arg level in phase 1 but a slightly different diet composition (Table 1). On day 21 (prior to diet change), one pig per pen (lowest BW of the two remaining pigs) was gavaged with lactulose and mannitol and blood was collected using previously described methods. After collecting blood, pigs were taken off phase 2 diets and fed a common diet for the remaining 21 d, with pigs and feeders weighed on day 42 to determine overall pig performance.

Morbidities and mortalities were recorded with removal dates and pig weights to adjust growth and feed intake data. The removal weight was added to the total pen weight, and the days the removed pig was in the pen were included in the total pig days. If a pig was removed during a phase transition, the pen’s starting weight for the next period was adjusted to reflect the remaining pigs’ growth. At the end of the study, two pens with no remaining pigs were excluded from the final phase and overall growth performance analysis.

Plasma samples were submitted to the University of Illinois (Champaign, IL) for the measurement of lactulose and mannitol concentration via high-performance liquid chromatography (Dionex ICS-3000/ICS-5000; Dionex Corp., Sunnyvale, CA). Measurements below the lower limit of detection (LOD; 0.1 µg/mL) were imputed by randomly sampling from a lognormal distribution starting at zero and truncated at the LOD. The parameters of the lognormal distribution were calculated from the original data, with samples below the LOD set equal to the LOD (Cohen and Ryan, 1989).

### Diet Analysis

Feed was manufactured at the Iowa State University Swine Nutrition farm feed mill. Feed samples were collected at the completion of mixing for each phase. Samples were then stored at −20 °C for subsequent analysis. Feed samples were ground to 1 mm particle size (Variable Speed Digital ED-5 Wiley Mill; Thomas Scientific, Swedesboro, NJ). Nitrogen and amino acid profiles were determined (N; Agriculture Experiment Station Chemical Laboratories, Columbia, MO) and crude protein was calculated as N × 6.25. Gross energy was determined in duplicate using an isoperibolic bomb calorimeter (model 6200; Parr Instrument Co., Moline, IL). Benzoic acid (6,318 kcal GE/kg) was used as the standard for calibration and was determined to contain 6,317 ± 10 kcal GE/kg.

### Statistical Analysis

Data were analyzed as a 2 × 4 factorial design using generalized linear models (Proc Glimmix and Proc Mixed, SAS 9.4, SAS Inst., Cary, NC) with pen as the experimental unit. The statistical model included the main effects of Arg-water level (0% vs. 8%) and Arg level in the feed (0.85, 0.95, 1.05, and 1.15 SID Arg:Lys) and the interaction between the two main effects. Growth parameters were analyzed separately for each phase. Ratio of absorbed lactulose to mannitol was log transferred to achieve normality. When fixed effects were a significant source of variation (*P* < 0.05) least squares means were separated using pairwise *t*-tests (PDIFF option, SAS 9.4, SAS Inst.) with a Tukey adjustment to account for multiple comparisons. Orthogonal polynomial contrasts were constructed to test linear and quadratic effect of increasing SID Arg:Lys in the diet. Results were considered significant at *P* ≤ 0.05 and a trend at *P* > 0.05 and *P* ≤ 0.10.

Data were analyzed using the following model:


$$\begin{array}{l} Y_{ijk}=\;\mu +\tau_{i}+\upsilon_{j}+{\left(\tau *\upsilon \right)}_{ij}+e_{ijk} \end{array}$$

where Y_ijkl_ is the observed value for the lth pen within the ith dietary Arg level and the jth Arg-water level; μ is the general mean: τ_i_ is the fixed effect of the ith dietary Arg level (i = 1 to 4); υ_j_ is the fixed effect of the jth Arg-water level (j = 1 or 2); (τ * υ) is the interaction between the ith dietary level and the jth water level of Arg; and e_ijk_ is the associated variance for the model Y_ijk_ of the lth pen (l = 1 through 80).

## Results

Pigs arrived from the sow facility with an average weaning weight of 5.06 ± 0.75 kg. At the end of the 42 d trial, pigs weighed approximately 19.82 ± 4.86 kg. Overall, the pigs had a 0.30 ± 0.10 kg ADG and a 0.66 ± 0.10 G:F. Dietary analysis (Table 2) showed similar gross energy within diets for each phase, total Arg increased as expected, with total Lys remaining similar. Crude protein values for diets were not as consistent as intended within a dietary phase. Analyzed Lys was higher in the phase 3 common diet than expected. The daily intake of Arg in grams is computed relative to feed intake and calculated SID Arg values (Table 2). As anticipated, pigs with higher dietary SID Arg:Lys ratios exhibited an increased daily Arg consumption.

**Table 2.** Analyzed gross energy (GE), total lysine (Lys), total arginine (Arg), and crude protein (CP) of dietary treatments on an as-fed basis by dietary phase^*^

	Dietary SID Arg:Lys			
	0.85	0.95	1.05	1.15
*GE, kcal/kg*				
Phase 1	4,003.8	4,001.7	3,998.7	4,004.9
Phase 2	4,063.9	4,009.2	4,017.3	4,033.2
Phase 3	4,006.1			
*Lys, %*				
Phase 1	1.78	1.78	1.67	1.58
Phase 2	1.63	1.60	1.58	1.61
Phase 3	1.79			
*Arg, %*				
Phase 1	1.44	1.62	1.80	1.89
Phase 2	1.30	1.42	1.54	1.83
Phase 3	1.26			
*CP* ^†^ *, %*				
Phase 1	21.43	22.64	22.89	22.88
Phase 2	21.07	21.35	21.89	22.41
Phase 3	19.66			
*SID Arg intake* ^‡^ *, g/d*				
Phase 1	0.74	0.83	0.92	1.01
Phase 2	3.74	4.18	4.62	5.06
Phase 3	8.91			

^*^Dietary phase 1 provided from days 0 to 7; phase 2 from 7 to 21; phase 3, a common diet, from days 21 to 42 of the experiment.

^†^Crude protein= %N × 6.25.

^‡^Predicted SID Arg intake based on calculated SID Arg and observed feed intake for each treatment/phase.

### Growth Performance

Growth responses exhibited considerable variability during the initial period, as evident from the negative G:F ratios reported in Table 3. Consequently, data were analyzed separately for each phase. The supplementation of Arg, whether in the water or the diet, did not yield any statistically significant effects.

**Table 3.** Main effects of feed- or water-based arginine on body weight (BW), average daily gain (ADG), average daily feed intake (ADFI), and gain to feed ratio (GF) for each phase and the overall 42-d trial

	Feed SID Arg:Lys^†^					Water, Arg^*^%			*P*-value			
	0.85	0.95	1.05	1.15	SEM	0%	8%	SEM	Water	Feed	Water × Feed	Linear feed^‡^
*BW, kg*												
Day 0	5.03	5.03	5.11	5.09	0.176	5.06	5.06	0.124	0.99	0.98	1.00	0.73
Day 7	4.54	4.76	4.88	4.71	0.174	4.69	4.75	0.122	0.73	0.57	0.58	0.41
Day 21	7.97	8.82	8.21	9.56	0.534	8.51	8.76	0.378	0.64	0.16	0.47	0.08
Day 42	18.47	19.67	19.43	21.90	1.092	19.70	20.03	0.762	0.76	0.16	0.16	0.04
*ADG, kg*												
Days 0 to 7	−0.06	−0.03	−0.05	−0.03	0.010	−0.05	−0.04	0.007	0.55	0.26	0.15	0.39
Days 7 to 21	0.25	0.29	0.24	0.30	0.028	0.28	0.27	0.019	0.68	0.34	0.70	0.37
Days 21 to 42	0.49	0.51	0.50	0.57	0.030	0.52	0.51	0.022	0.80	0.27	0.19	0.09
Days 0-42	0.28	0.31	0.28	0.34	0.023	0.31	0.30	0.016	0.74	0.14	0.36	0.11
*ADFI, kg*												
Days 0 to 7	0.05	0.05	0.05	0.06	0.006	0.05	0.05	0.004	0.30	0.53	0.06	0.27
Days 7 to 21	0.28	0.34	0.30	0.34	0.030	0.30	0.33	0.021	0.39	0.33	0.63	0.24
Days 21 to 42	0.72	0.76	0.74	0.84	0.040	0.76	0.77	0.028	0.64	0.18	0.37	0.06
Days 0 to 42	0.43	0.47	0.43	0.49	0.028	0.46	0.45	0.020	0.98	0.27	0.45	0.23
*G:F*												
Days 0 to 7	−1.78	−0.95	−1.72	−1.04	0.336	−1.62	−1.12	0.232	0.14	0.16	0.20	0.32
Days 7 to 21	0.82	0.89	0.84	0.91	0.032	0.89	0.84	0.023	0.14	0.13	0.14	0.10
Days 21 to 42	0.70	0.69	0.70	0.69	0.014	0.70	0.69	0.010	0.82	0.85	0.90	0.82
Days 0 to 42	0.63	0.67	0.65	0.69	0.025	0.66	0.65	0.018	0.61	0.34	0.36	0.12

^*^0% or 8% arginine as the free base stock solution prior to 1:128 dilution into the water system.

^†^SID, standardized ileal digestible; Arg, arginine; Lys, Lysine.

^‡^Orthogonal polynomial contrast, quadratic effect insignificant for all parameters.

Although a significant linear trend (*P* = 0.04) was observed for dietary Arg’s influence on final BW at day 42, this effect did not translate into discernible differences between the various dietary treatments. Additionally, a trend (*P* = 0.09) was detected, suggesting a linear impact of dietary Arg on the ADG of pens during the third period, encompassing days 21 to 42.

### Intestinal Absorption and Morphology

Dietary and waterborne Arg supplementation displayed no discernible effects on lactulose absorption compared to mannitol on both days 7 and 21 (*P* > 0.1, Table 4). Furthermore, no differences were observed in the villous height, crypt depth, or the villous:crypt ratio in ileum tissues collected on day 7 postwean.

**Table 4.** Main effects of feed- or water-based arginine on gastrointestinal structure and permeability

	Feed SID Arg:Lys					Water, Arg %			*P*-value			
	0.85	0.95	1.05	1.15	SEM	0%	8%	SEM	Water	Feed	Water × Feed	Linear feed ^†^
*Day 7*												
Lactulose:mannitol^*^	0.02	0.06	0.03	0.04	0.065	0.03	0.03	0.023	0.81	0.18	0.98	0.35
Villous, µm	273.24	271.48	253.24	283.25	13.049	270.62	282.33	9.105	0.89	0.33	0.27	0.48
Crypt, µm	122.18	123.11	118.23	126.02	4.432	121.42	124.92	3.092	0.83	0.58	1.00	0.43
Villous:Crypt	2.24	2.23	2.17	2.26	0.095	2.25	2.27	0.067	0.90	0.88	0.17	0.48
*Day 21*												
Lactulose:mannitol^*^	0.09	0.15	0.12	0.10	0.180	0.09	0.21	0.090	0.21	0.43	0.64	0.35

^*^Pigs were orally gavaged with a lactulose-mannitol solution, 500 mg/kg of lactulose, and 50 mg/kg body weight of mannitol. Blood samples were collected approximately four hours later to determine the lactulose:mannitol in the blood plasma. Data were log-transformed to achieve normality and then back-transformed.

^†^Orthogonal polynomial contrast, quadratic effect insignificant for all parameters.

## Discussion

The current experiment was conducted to understand the impact of increasing dietary SID Arg:Lys and water supplementation with Arg based on previous studies (Greiner et. al., 2023). The pigs in the current experiment were naturally challenged with enteric disease (*Rotoviral enteritis*, *Coccidiosis*, and *Escherichia coli*) within the first week of arrival. *Escherichia coli* and *Rotavirus* infection lead to osmotic movement of fluid into the lumen leading to malabsorption and diarrhea. These pathogens are common in nursery pigs and explain the severe weight loss in the first week postwean.

Arginine supplementation over NRC recommendations has been successfully utilized to improve growth performance, meat quality, and immunity of broilers (Hassan et al., 2021). In pigs it has been shown to alter the catabolism of fat and amino acids in the body and thus enhance protein synthesis in skeletal muscle (He et al., 2009). Previously, it has been reported that increasing dietary SID Arg improved final BW, and water-based Arg improved intestinal permeability measurements (8% stock solution, Greiner et al., 2023). Therefore, the objective of this experiment was to further explore the impact of water-based Arg at an 8% stock solution (0.0625% final concentration consumed by the pig) and the impact of increasing SID Arg:Lys. In this study, although the pigs were already infected with pathogens before we began Arg supplementation, preventing it from providing immediate protective benefits, the supplement did show potential in aiding recovery from such exposures. This potential was indicated by improvements in ADG and BW. Supplemental Arg led to a steady increase in the final BW of pigs, which aligns with findings from Greiner et al. (2023), and may indicate that the requirement for Arg may be higher than current recommendations.

Arginine serves as a precursor for the synthesis of NO, a crucial signaling molecule involved in several physiological processes. NO acts as a vasodilator and promotes angiogenesis, enhances blood flow, and facilitates the development of brown fat, as documented by Dai et al. (2013). These effects likely enhance blood flow and nutrient distribution, supporting growth by promoting tissue repair and protein synthesis in skeletal muscle. Similarly, in the state of enteric disease challenge, Arg may enhance blood flow to the intestines to allow for improved nutrient assimilation, which may contribute to the growth response observed in the current study.

The need for Arg by macrophages for NO synthesis may also increase Arg demand during disease states. Additionally, increased NO levels contribute to the development of brown adipose tissue, known for its role in energy expenditure and thermogenesis, which might explain previously observed improvements in ADG and feed efficiency during periods of heat stress (Yun et al., 2020). Furthermore, due to these mechanisms, Arg supplementation has also been recommended for treating human metabolic syndromes (Dai et al., 2013). Although more research is needed to fully understand how supplemental Arg benefits growth and health by modulating NO levels and affecting various physiological pathways, it remains a promising option for enhancing growth and health outcomes in pigs. The observed improvement in feed intake after pigs were switched to a common diet is not fully understood, but it may simply be due to their increased BW prior to the diet change.

Lactulose and mannitol absorption test has been considered the gold standard in intestinal permeability research in humans (Ménard et al., 2010; Galipeau and Verdu, 2016) and has been the most reliable indicator of intestinal permeability in patients (Vilela et al., 2008). The ratio of lactulose to mannitol in circulation provides a measure of intestinal barrier integrity. Impaired tight cell junctions can lead to paracellular migration of larger molecules, such as lactulose, into the bloodstream. Consequently, the ratio of absorbed lactulose to mannitol offers researchers insight into the extent of intestinal permeability, with a higher ratio generally indicating a more permeable epithelial barrier (Fleming et al., 1996; Hu et al., 2023). Previous research by Greiner et al. (2023) demonstrated that final concentrations of 0.03125% and 0.0625% Arg, derived from 4% and 8% water-based Arg stock solutions, respectively, significantly reduced serum lactulose ratios compared to a group without water supplementation. However, in our current study, an 8% stock solution (0.0625% final concentration consumed by the pig) did not reduce intestinal permeability, potentially due to heightened disease pressure compared to the Greiner et al. (2023) study, where pigs experienced low natural disease pressure. The authors recognize that the lactulose: mannitol values are lower than those reported in Greiner et al. (2023) which could suggest generally lower intestinal permeability of the pigs in the current trial. However, it was observed that the pigs in the present study had a high incidence of scouring due to the enteric challenge, thus the osmotic flux of fluid into the lumen may have partially inhibited paracellular movement of the lactulose and mannitol molecules.

Consistent with Greiner et al. (2023), feed-based Arg did not improve measurements of intestinal permeability in the current study. Yet, other studies noted that supplemental Arg has demonstrated the ability to maintain barrier function in mice (Viana et al., 2010), decrease permeability during hypothermia (Costa et al., 2014), and reduce intestinal inflammation during pathogen challenge (*Escherichia coli*, Liu et al., 2008). Variations in results may be attributed to factors such as health stress, timing of intestinal measurements, or methodological differences.

In the present study, feed and/or water did not influence intestinal morphology, unlike findings by Greiner et al. (2023), who observed that water supplementation influenced crypt depth and V:C. Arg has been shown previously to protect the intestine from morphological damage, where dietary supplementation with 0.5% and 1% Arg alleviated morphological alterations during an LPS challenge (Liu et al., 2008). While LPS is a known model for intestinal injury (Mercer et al., 1996), it is possible that the natural health challenge in the current study was much stronger, therefore masking the potential health benefits of Arg. Although supplemental Arg supported improved piglet growth, the strength of the health challenge may have restricted Arg ability to elicit improvements in intestinal morphology as previously observed (Greiner et al., 2023).

In conclusion, when pigs faced significant enteric challenges upon arrival, no benefits of Arg supplementation (water nor diet) were discovered on the intestinal permeability or structure with the parameters measured. Together, the improved growth responses observed in the current study and by Greiner et al. (2023) suggest that the SID Arg:Lys requirement may be higher than current recommendations. Further research is warranted to define the ideal SID Arg:Lys ratio to maximize growth and potentially health, as these could not be determined with the current data.
